# Cardiovascular Health of Construction Workers in Hong Kong: A Cross-Sectional Study

**DOI:** 10.3390/ijerph15061251

**Published:** 2018-06-12

**Authors:** Joanne Wai-Yee Chung, Bonny Yee-Man Wong, Vincent Chun-Man Yan, Louisa Ming-Yan Chung, Henry Chi-Fuk So, Albert Chan

**Affiliations:** 1 Department of Health and Physical Education, The Education University of Hong Kong, 10 Lo Ping Road, Tai Po, New Territories, Hon Kong, China; bymwong@eduhk.hk (B.Y.-M.W.); cmyan@eduhk.hk (V.C.-M.Y.); chungmy@eduhk.hk (L.M.-Y.C.); 2 Department of Mathematics and Information Technology, The Education University of Hong Kong, 10 Lo Ping Road, Tai Po, New Territories, Hong Kong, China; hcfso@eduhk.hk; 3 Department of Building and Real Estate, Polytechnic University, 11 Yuk Choi Rd, Hung Hom, Hong Kong, China; albert.chan@polyu.edu.hk

**Keywords:** construction industry, heart health, lifestyle behaviors, healthy eating, physical activity

## Abstract

Background: Given a shortage of construction workers, it is important to develop strategies to avoid early retirement caused by cardiovascular diseases in Hong Kong. Objectives: (1) to describe the cardiovascular health of construction workers in Hong Kong, (2) to examine the demographic differences in cardiovascular health, and (3) to examine the association between health behaviors and cardiovascular health factors. Methods: 626 registered construction workers were included in the analysis. Blood chemistry, blood pressure, weight, and height were measured. Face-to-face questionnaire interviews for health behaviors were conducted. Results: Approximately two-thirds of the construction workers achieved only three out of the seven “ideal” cardiovascular health metrics. The younger, more educated, and female subjects had better cardiovascular health scores than the older, less educated, and male counterparts. Fish and seafood consumption was associated with (1) ideal weight status and (2) ideal cholesterol level, whereas less soft drink consumption was associated with ideal cholesterol level. Conclusions: The findings highlighted the importance of promoting cardiovascular health in the construction industry. This study provided some insights for future interventions, which should include increasing fish and seafood intake, decreasing soft drink consumption, and enhancing the health literacy amongst older, less educated, and male construction workers.

## 1. Introduction

Construction work is highly demanding, because workers are incessantly exposed to harsh environments filled with fumes, dust, heat, and moisture [1]. Cardiovascular diseases, following musculoskeletal diseases, are one of the major causes for early retirement among these workers, because of the “healthy worker effect” [2,3,4]. Risks for poor cardiovascular health were more prevalent among construction workers than those in other occupations. In one study, construction workers were 15% and 9% more likely to have hypertension and diabetes, respectively, when compared with workers in the service sector (OR_hypertension_: 1.15, 95% confidence interval (CI): 1.11–1.18; and OR_diabetes_: 1.09, 95% CI: 1.02–1.17) [5]. In another study, construction workers were 56% more likely to be obese than white-collar workers [6]. Seventy-one percent of construction workers were either overweight or obese, compared with 67% of all industries [2]. When compared with office clerks and professionals, construction workers were more likely to have metabolic risk factors (raised blood triglycerides, high-density-lipoprotein (HDL) cholesterol, fasting glucose, central obesity, and hypertension) (OR: 1.62, 95% CI: 1.03–2.56) [7]. A related finding also revealed that blue-collar workers, to which construction workers belong, had 4.3 times higher risk of a first event of non-fatal acute ischemic heart disease than white-collar workers [8]. Similar to Western countries, Hong Kong’s construction industry suffers from a shortage of construction workers and a depleting aging workforce. To solve these problems, workers’ health behaviors and cardiovascular health factors, apart from the workplace as pinpointed by the World Health Organization [9], may have to be looked into more closely in order to avoid early retirement caused by the diseases and to improve the productivity of the existing workforce.

Regarding the cardiovascular risks of construction workers in Hong Kong, so far there are only two research studies, one with a focus on musculoskeletal pain [10] and the other on comparing the cardiopulmonary risk in different occupations [7]. In other words, the cardiovascular health of construction workers has not been thoroughly evaluated; it is essential to have this systematic assessment done in order to monitor the trends, to make studies comparable, and hence, to develop long-term strategies to promote cardiovascular health to reduce premature retirement in the construction industry. At present, in the absence of such an assessment, it is almost impossible to prioritize effectively for whom and what future interventions should be targeted.

To develop such an assessment, the American model can be adopted. The American Heart Association has established “ideal cardiovascular health” metrics to monitor and quantify Americans’ cardiovascular health and track their progress towards the national goals set under the Healthy People 2020 by the United States [11]. The “ideal cardiovascular health” metrics include three health behaviors (physical activity, absence of smoking, and dietary habits) and four cardiovascular health factors (normal weight status, serum total cholesterol <200 mg/dL, blood pressure <120/<80 mm Hg, and absence of diabetes) [11]. The underlying mechanism is that unhealthy behavioral risk factors (physical inactivity, unhealthy diets, and smoking) would lead to hypertension, diabetes, high blood cholesterol, and obesity—“intermediate risk factors”, which can indicate increased the risk of developing cardiovascular diseases [12]. It is found that modifiable lifestyle behaviors, for example, unhealthy eating, smoking, and lack of physical activity (PA), have been consistently associated with cardiovascular diseases [11,13,14]. Adopting healthy lifestyle behaviors can be a strategy to reduce the risk of cardiovascular diseases by improving cardiovascular health factors (e.g., blood pressure, blood glucose, blood cholesterol, and weight status). However, most studies on cardiovascular health in construction workers were descriptive studies [2,5,6,7,10,15]. Construction workers had a high level of occupational PA and were presumably fit, but they were found to have higher risks for poor cardiovascular health than workers in other industries [2,5,7,16], suggesting that PA may not sufficiently protect their cardiovascular health. The associations between healthy behaviors and cardiovascular health factors are worth investigating so that specific health behaviors, other than PA, that may help reduce the risks of cardiovascular diseases could be identified in this specific group.

There have not been any studies using lifestyle interventions specifically for construction workers until the recent decade [17,18]. One lifestyle intervention reported beneficial effects on smoking, fruit and snack intake, and weight status, but no effect on other cardiovascular health factors (e.g., cholesterol and blood glucose) and health behavior (e.g., physical activity) [19,20]. Another intervention aimed to improve male construction workers’ weight status, blood pressure, and cholesterol by changing physical activity and dietary habits [21]. Only weight status was improved, and consumption of sweetened beverage was reduced [21]. Multiple behavior interventions have been found to be less effective in changing subjects’ lifestyle behaviors than single behavior interventions, as changes in multiple behaviors might make the subjects feel overwhelmed [22]. Instead of addressing common healthy lifestyle behaviors, intervention should be tailor-made and evidence-based to address specific health behaviors and cardiovascular health factors. Therefore, investigating the association between health behaviors and cardiovascular health factors among construction workers could shed the light on future tailor-made interventions for them.

With limited previous studies, the systematic profile of Hong Kong construction workers with cardiovascular health remains unclear. The association between their health behaviors and cardiovascular health factors could provide insight into future strategies to prevent non-communicable diseases (e.g., cardiovascular diseases), reduce premature retirement, and improve the productivity of the existing workforce. Therefore, the objectives of this paper were (1) to describe the cardiovascular health of the construction workers in Hong Kong, using the “ideal cardiovascular health” metrics of the American Heart Association [11]; (2) to examine the workers’ demographic differences in the cardiovascular health metrics; and (3) to examine the association between their health behaviors (physical activity, absence of smoking, and healthy eating) and cardiovascular health factors (weight status, blood pressure, blood glucose, and blood cholesterol).

## 2. Materials and Methods

### 2.1. Subjects and Procedures

The subjects were recruited by convenience sampling. From November 2014 to August 2016, the Construction Industry Council invited all construction companies in Hong Kong to join the program called “Pilot Medical Examination Scheme”. Forty-six construction companies agreed to join the program and supported their registered construction workers to participate. As a result, a total of 955 registered construction workers from 46 construction sites (28% in the New Territories, 39% in Kowloon, and 32% in Hong Kong Island [Hong Kong is made up of these three areas]) were recruited. The research team visited each construction site during lunch breaks and met up with each worker for about 15 min. The inclusion criterion was that they were registered workers under the Construction Worker Registration System in Hong Kong. All construction workers in construction sites were registered and hence, we had access to the entire construction worker population. Written consent was obtained from each participant before the research team measured his/her weight and height and carried out the face-to-face questionnaire interview. His/her blood sample and blood pressure information were taken on-site by the staff members from a clinic. Ethical approval was obtained from the Research and Development Office, the Education University of Hong Kong, Hong Kong.

Only 626 subjects provided full data and hence, were included in the analysis. Missing data came from those who did not fast on the day of data collection and had only received random blood glucose tests (*n* = 329). There was no significant difference in the demographic background between the included and the excluded subjects (*p* > 0.05) [23,24].

In Hong Kong, there were approximately 450,757 registered construction workers in 2014 [25]. Post-hoc power analysis showed that with the current sample size of 626, the confidence level was 95% and margin of error was 3.9 [26], and current sample size had sufficient power.

### 2.2. Dependent Variables: Anthropometric Measures and Blood Tests (Clinical Examination)

The workers’ weight and height were measured on-site by research assistants. The body mass index (BMI (kg/m^2)^) for each worker was then computed and categorized into three groups: underweight (BMI < 18 kg/m^2^), normal weight (BMI = 18–22.9 kg/m^2^), and overweight (BMI ≥ 23 kg/m^2^). The measurement of blood sample and pressure were sorted into three categories: normal (<120 mm Hg/<80 mm Hg), pre-hypertension (systolic blood pressure (SBP) = 120–139 mm Hg or diastolic blood pressure (DBP) = 80–89 mm Hg), and hypertension (SBP ≥ 140 mm Hg or DBP ≥ 90 mm Hg). The clinic performed the blood sample test to examine the blood glucose (fasting or random) and the total cholesterol level. The automatic integrated chemical analyzer (Ortho-Clinical Diagnostics VITROS5.1 FS Chemistry System (Ortho-Clinical Diagnositics, Rochester, NY, US) [27]) was used for automatic blood analysis. The estimates of variabilities were 0.115 mmol/L and 0.112 mmol/L for the glucose and cholesterol test, respectively [28]. The laboratory is certificated by the Hong Kong Institute of Medical Science Quality Assurance Program. Only the workers who had the fasting blood glucose measured were included in the analysis. The fasting blood glucose was classified as normal (<5.6 mmol/L converted as <100 mg/dL), glucose impaired tolerance (5.6–6.9 mmol/L converted as 100–125 mg/dL), and diabetes (≥7.0 mmol/L converted as ≥126 mg/dL). Similarly, the total cholesterol level was categorized as normal (<200 mg/dL), borderline high (200–239 mg/dL), and high (≥240 mg/dL).

For data analysis, cardiovascular health factors were classified according to the American Heart Association into “ideal” versus “undesired”, which represented normal and higher than normal ranges, respectively [11]. Using this guideline, the “ideal” versus “undesired” classifications for the weight status were BMI < 23 kg/m^2^ versus BMI ≥ 23 kg/m^2^, blood pressure were <120 mm Hg/<80 mm Hg versus ≥120 mm Hg/≥80 mm Hg, fasting blood glucose were <5.6 mmol/L converted as <100 mg/dL versus ≥5.6 mmol/L converted as ≥100 mg/dL, and total cholesterol were <200 mg/dL versus ≥200 mg/dL. The total number of ideal cardiovascular health factors was then calculated (range: 0–4).

### 2.3. Independent Variables: Lifestyle Behaviors (Questionnaire)

The questionnaire comprised of questions regarding health behaviors of the subjects, including leisure-time PA, smoking status, and diet pattern, which were adopted from a validated dietary questionnaire [29] (Table A1).

To obtain information on “ideal” or “undesired” behaviors on leisure-time PA, the question asked was, “In the last month, how often did you participate in sports?”, with the response in a nine-point Likert scale. Subjects were then asked a follow-up question; “How much time did you spend in sports on the day that you participated in sports?”, with the response in a six-point Likert scale. Following this, they were also asked, “What sports did you play?”, with seven response options. This question was used to divide the PAs into moderate PA and light PA. Given that the intensity was not asked, to be conservative, “ball games”, “cycling”, “running”, “jogging”, “swimming”, and “hiking” were considered as at least moderate physical activity, whereas “other”, commonly including yoga, tai chi, and qi-qigong, was considered as light physical activity [30]. For the duration of PA, the responses were recoded as 15 min, 30 min, 1 h, 2 h, 3 h, and 4 h. In terms of the frequency of PA, the responses were recoded as 0 days a week, 0.25 days a week, one day a week, two days a week, three days a week, four days a week, five days a week, six days a week, and seven days a week. Total duration of PA was computed by multiplying the frequency with the duration. The seven-day test-retest reliability of these three questions were 0.981, 0.931, and 0.943 (*n* = 16), correspondingly. Leisure-time PA was considered as “ideal” if the subject had 150 minutes or more of moderate PA per week, and as “undesired” if he/she did less than 150 min per week.

To evaluate smoking, the subjects were asked the question, “How often did you smoke in the last month?”, with the response in a nine-point Likert scale. They were then asked a follow-up question; “How much did you smoke on the day that you smoked?”, with a six-point Likert scaled response. Test-retest reliability of these two questions was one. Those subjects who selected smoking frequency as “never” and number of cigarettes smoked on that day as “none” were considered as non-smokers. Smoking status was classified as “ideal” for a non-smoker (i.e., no cigarettes smoked) and as “undesired” for a smoker smoking one or more cigarettes in the last 30 days.

Three diet consumption patterns were designed: (1) fruits and vegetables, (2) fish and seafood, and (3) soft drinks. To reveal the frequency of certain food intake, the subjects were asked the question, “How often did you eat/drink the following food/beverage (vegetables, fruits, fish, seafood, and soft drinks) in the last month?”, with the response in a nine-point Likert scale. Their seven-day test-retest reliability ranged from 0.856 (seafood and fish) to 0.99 (vegetable) (*n* = 16). Then, they would be asked a follow-up question on the amount consumed; “How much did you eat/drink the above food/beverage on the day that you ate/drank it?”, with the response in a eight-point Likert scale. Their seven-day test-retest reliability ranged from 0.782 (seafood and fish) to 1.00 (soft drinks) (*n* = 16). The above questions were to obtain the frequency and the respective amount of the food/drinks consumed in a subject’s dietary patterns. In terms of the frequency, the responses were recoded as 0 days a week, 0.25 days a week, one day a week, two days a week, three days a week, four days a week, five days a week, six days a week, and seven days a week. For the amount of food consumed, the responses were recoded as 0, 1, 2, 3, 4, 5, 6, and 7 serving(s). The total amount of the food/drink consumed was then computed. Fruit and vegetable consumption were defined as “ideal” if the diet included 4.5 servings (cups) or more of fruits and vegetables per day, and as “undesired” if less than 4.5 cups per day. According to the American Heart Association, fish consumption should be at least two servings per week [11]. As the study was designed to develop a health profile for Hong Kong construction workers, the consumption of fish was expanded to include seafood. That said, the majority of seafood consumption in Hong Kong was fish [31], and hence such a measure reasonably assessed the fish consumption. The fish and seafood consumption was separated into two groups: the “ideal” with two or more servings per week, and the “undesired” with less than two servings per week. This study included only soft drink consumption, rather than all sugar-sweetened beverages, as suggested by the American Heart Association [11]. Soft drink consumption was classified as “ideal” if the subject took less than 36 oz of soft drinks per week, and as “undesired” if he/she consumed more than 36 oz per week. From the responses of each subject, the subjects’ dietary behaviors were considered as “ideal” if they had ideal responses to the three aforementioned items, or else their behaviors would be considered as “undesired”. The total number (range: 0–3) of “ideal” health behaviors (i.e., PA, smoking, and dietary behaviors) was then counted.

#### Cardiovascular Health Score

Cardiovascular health score was based on the American Heart Association’s cardiovascular metrics, which measured seven items, namely, weight status, blood pressure, blood glucose level, cholesterol level, PA, dietary behaviors, and smoking [11]. The score, ranging from 0 to 7, is the sum of the total number of “ideal” conditions for the seven metrics to indicate the cardiovascular health of a participant.

### 2.4. Covariates: Demographic Characteristics (Questionnaire)

The questionnaire also comprised of questions regarding demographic characteristics of the subjects. The covariates were gender (males and females), age (20–29, 30–39, 40–49, 50–59), education (no formal education/primary education, junior secondary education, senior secondary education, and post-secondary education), and ethnicity (Chinese: born in Hong Kong or have lived in Hong Kong for more than 7 years, Chinese: from Mainland, Minority).

### 2.5. Statistical Methods

Frequency and percentage were used to summarize the construction workers’ cardiovascular health factors (blood pressure, glucose level, cholesterol level, and weight status) and health behaviors (smoking, dietary behaviors, and leisure-time PA), whereas mean and standard deviation were used to describe cardiovascular health score. For objective 2, Chi-square tests were performed to examine the association between demographic characteristics (age, gender, education, and race) and the individual cardiovascular health factors and health behaviors (binary variables). Given that cardiovascular health score, and overall cardiovascular health factors and health behaviors, were normally distributed, T-tests and one-way analysis of variance (ANOVAs) were performed to compare the cardiovascular health score, and overall cardiovascular health factors and health behaviors by demographic characteristics (age, gender, education, and race). For objective 3, multilevel logistic regressions were performed to regress the cardiovascular health factors on health behaviors, adjusting for age, gender, education, and ethnicity and the cluster effect of the construction sites. All statistical analyses were performed using STATA 13 (StataCorp LP, College Station, TX, USA).

## 3. Results

### 3.1. Demographic Characteristics of the Sample

Out of the 626 subjects, 90% of them were male with 67% aged 40 or above (Table 1). In terms of ethnicity, approximately 84% were born in Hong Kong and 12% were from Mainland China, whilst the remaining (4%) were from Nepal, Pakistan, or other countries. About 20% of them had no formal education or only primary education. The percentages of subjects who had junior secondary (Form 3), senior secondary (Form 7 in the old education system or Form 6 in the new system), and post-secondary education (e.g., diploma, associate, or degree) were 40%, 27%, and 11%, respectively. The sample was representative of the construction workers in Hong Kong, with age and gender distribution consistent with government data for 2015 [32].

### 3.2. Cardiovascular Health

As shown in Table 1, 38% of the subjects had ideal weight status, 68% had ideal total blood cholesterol level, 19% had ideal blood pressure, and 92% had ideal fasting blood glucose level.

Regarding health behaviors, 60% of the subjects were non-smokers and 28% engaged in an ideal amount of PA. In terms of the consumption of fruits and vegetables, soft drinks, and fish and seafood, 8%, 73%, and 59%, respectively, of the subjects were considered to be in an ideal state.

The average cardiovascular health score (range: 0–7) was 3.07 (SD = 1.14). The distribution of the health scores among the subjects from score 0 to score 7 was 0.5%, 6.7%, 25.1%, 32.7%, 24.4%, 9.3%, 1.1%, and 0.2%, respectively. The mean number of ideal health behaviors (range: 0–3) was 0.91 (SD = 0.70) and the distribution of scores from 0 to 3 was 28.4%, 52.6%, 18.2%, and 0.8%, respectively. The mean number of cardiovascular health factors (range: 0–4) was 2.16 (SD = 0.91) and the distribution of scores from 0 to 4 was 2.4%, 20.9%, 42.3%, 27.3%, and 7.0%, respectively.

### 3.3. Demographic Differences in Cardiovascular Health

As shown in Table 2, there were no significant differences in the weight status, total cholesterol, blood pressure, and fasting blood glucose across the gender, educational, and ethnic groups. In contrast, there were significant differences in the above-mentioned items across specific age groups. More subjects aged 20–29 had ideal weight status and cholesterol level when compared with those in other age groups (*p* = 0.01 and *p* < 0.001, respectively), whereas more subjects in the age groups of 20–29 and 30–39 had ideal fasting blood glucose when compared with those in the other age groups (*p* < 0.001).

There were no significant differences in the fruit and vegetable consumption among the gender, age, ethnic, and education groups (Table 3). However, significant differences were found in other lifestyle behaviors across the gender, age, ethnic, and education groups. More male subjects were smokers (*p* < 0.001) and engaged in more leisure-time PA than the females (*p* = 0.007). In general, the percentage of subjects achieving the ideal amount of leisure-time PA decreased with age (*p* < 0.001), whereas the percentage of the ideal amount of fish and seafood consumed by the subjects increased with age (*p* < 0.001). The Chinese who were born in Hong Kong or lived in Hong Kong for more than seven years were more physically active when compared with the new Chinese migrants from the Mainland (*p* = 0.028). Besides, the Chinese subjects consumed more seafood and fish than other ethnic groups (*p* = 0.002). Regarding educational levels, they were positively correlated with the percentage of the ideal amount of leisure-time PA (*p* < 0.001), but negatively correlated with the percentage of the ideal amount of fish and seafood (*p* = 0.002) and soft drink consumed (*p* = 0.031).

In terms of overall cardiovascular health, as shown in Table 4, the younger, more educated, and female subjects had higher cardiovascular health scores than the older, less educated, and male subjects (*p* = 0.003, *p* < 0.001, *p* < 0.001, respectively). Similarly, the younger, more educated, and female subjects had more ideal lifestyle behaviors than their older, less educated, and male counterparts (*p* < 0.001, *p* = 0.016, *p* < 0.001 respectively). With respect to the cardiovascular health outcomes, significant differences occurred across different age groups (*p* < 0.001); the subjects aged 20–29 had more ideal cardiovascular health outcomes than those aged 50–59.

### 3.4. Associations between Clinical Test Results and Lifestyle Behaviors

Only a few lifestyle behaviors were related to the cardiovascular health outcomes (Table 5). The non-smokers were more likely to have ideal weight status when compared with the smokers (adjusted OR: 1.36, 95% CI: 1.02–1.81, *p* = 0.03). The consumption of fish and seafood was associated with ideal weight status and total cholesterol level (adjusted ORweight: 1.34, 95%: CI: 1.02–1.76, *p* = 0.04; adjusted ORchol: 1.35, 95% CI: 1.002–1.82, *p* = 0.048). The subjects who drank less soft drinks were more likely to have ideal total cholesterol when compared with those who drank excessively (adjusted OR: 1.48, 95%: 1.06–2.07, *p* = 0.02).

## 4. Discussion

About two-thirds of the subjects only achieved three out of the seven “ideal” cardiovascular health indicators in this study. The cardiovascular health metrics have not been used in the construction industry. Few existing studies examined cardiovascular health factors and health behaviors (e.g., hypertension, diabetes, serum cholesterol, obesity, waist circumference, and smoking); however, they focused more on cardiovascular health factors and described them individually, instead of quantifying the number of risk/protective factors [3,15,33]. In the recent decade, the American Heart Association established cardiovascular health metrics to monitor both health behaviors and cardiovascular health factors in order to reduce the risk of cardiovascular diseases [11]. Recently, Tin and her colleagues evaluated cardiovascular health and health behaviors comprehensively [7], but the risk for cardiovascular diseases still was not quantified as the present study has done. Quantifying cardiovascular health provided a clearer picture of construction workers’ risk of developing cardiovascular diseases. The present study justified the urge to develop strategies to promote cardiovascular health among construction workers by finding that two-thirds of workers had at least four risk factors of cardiovascular diseases.

Despite that the cardiovascular health metrics for the population in Hong Kong are not available, the prevalence of some health behaviors and cardiovascular health factors are available to be compared with construction workers. Sixty-two percent of the construction workers were overweight or obese as compared with 39% of the adults in Hong Kong [34]. Eighty-one percent of the subjects had hypertension/pre-hypertension. The corresponding figure for adults in Hong Kong was 32% [35]. The percentages of the subjects who smoked, were physically inactive, and consumed fruits and vegetables less than recommended were 40%, 72%, and 93%, correspondingly. The corresponding figures for adults in Hong Kong were 11%, 52%, and 79% [34]. Compared with the general adult population, hypertension, obesity, smoking, lack of physical activity, and low fruit and vegetable intake were more prevalent in construction workers, probably because of lower socioeconomic status [16].

Consistent with previous studies [2,3,6,33], the prevalence of pre-hypertension/hypertension (81%), borderline high blood cholesterol/hypercholesterolemia (32%), and overweight/obesity (62%) was high in construction workers in Hong Kong. Many studies have already demonstrated that the “healthy worker effect” is paradoxical; cardiovascular diseases such as ischaemic heart disease, heart failure, and hypertension are permanent disabilities that forced workers to retire early [3,4,36,37] or caused them to die prematurely [36,38]. Moreover, cardiovascular disease was the second leading cause of permanent disability and the leading cause of premature death among construction workers [4,38]. Hence, promoting cardiovascular health in the construction industry will enhance the sustainability of the existing workforce.

Younger, higher educated, and female workers were found to have more health behaviors. They were found to have higher health literacy, and hence might have greater awareness of health problems and more healthy lifestyles [39]. Future interventions should enhance the health literacy of construction workers, particularly the older, less educated, and male workers.

This study found that the subjects who consumed fish and seafood were more likely to have a healthier weight status than those who did not. This is consistent with the literature suggesting that fish and seafood consumption improved weight status, possibly through reduction in body fat mass, and satiety regulation and lipid oxidation stimulated by *n*−3 polyunsaturated fatty acid (PUFA) [40,41]. Moreover, the construction workers who consumed more fish and seafood were more likely to have lower serum cholesterol levels. Some previous studies suggested that phospholipids and fish protein consumption could lower serum cholesterol levels, possibly by reducing serum and liver cholesterol levels, improving lipid metabolism, inhibiting cholesterol and bile acid absorption, and enhancing cholesterol catabolism in the liver [40,42,43,44]. This was probably the reason why the United States’ recent dietary recommendation suggested two servings (eight ounces) of seafood per week [45]. This study could not establish the causal relationship between fish and seafood intake and low serum cholesterol and weight status due to the cross-sectional study design. Longitudinal studies should be conducted to confirm whether promoting fish and seafood intake in the construction industry could be one of the effective strategies to enhance cardiovascular health among construction workers.

The subjects who consumed less soft drinks were found to have lower serum cholesterol level when compared with those with excessive consumption. An intervention study showed that reduction in soft drink consumed lowered the total serum cholesterol level in school-aged children [46]. Similarly, another study demonstrated that drinking colas increased total serum cholesterol [47]. Besides, some other studies also identified related associations: drinking soft drinks was related to lower high-density-lipoprotein (HDL) cholesterol (good cholesterol) and higher triglycerides, and low-density-lipoprotein (LDL) and very-low-density-lipoprotein (VLDL) cholesterol, a precursor of low-density-lipoprotein [48,49,50]. Such a change in serum lipid profiles may have resulted from the alteration of lipid metabolism and profiles in liver and serum by the added sugar (e.g., fructose) and acid in soft drinks [50,51,52]. As recommended by the American Heart Association, sugar-sweetened beverages should be reduced to less than 36 oz per week [11], future intervention should consider promoting such a dietary recommendation in order to improve cardiovascular health in construction workers.

Hypertension was prevalent in construction workers (81% of construction workers versus 32% of general population); however, there was no association between health behaviors and hypertension. In recent years, evidence has suggested that leisure time PA decreased the risk of cardiovascular diseases, whereas occupational PA increased such risk [53,54]. It was reported that high occupational PA and lifting heavy loads increased ambulatory blood pressure, whereas leisure-time PA decreased ambulatory blood pressure [54]. Because no information on occupational PA was available, excluding occupational PA in analysis may lead to null association between health behaviors and hypertension. Similarly, occupational PA could diminish the beneficial effect of leisure-time PA on the resting heart rates, possibly because of imbalanced autonomic regulation caused by restricted, heavy activities related to productivity with limited time for recovery [53]. Future studies should consider occupational PA to gain a better understanding on health behaviors and cardiovascular health factor among construction workers.

Our results showed that absence of smoking was associated with normal weight status among construction workers. In current literature, smoking was associated with lower BMI possibly because of the effect of nicotine on metabolism [55,56]. However, in-depth analysis showed that heavy smokers were likely to have greater BMI than non-smokers and light smokers possibly because of tendency of adapt multiple unhealthy behaviors [57]. In the present study, 90% of smoking participants smoked five cigarettes or more (the highest amount in the response options). As a result of the question design, classification of light (defined as consuming 10 cigarettes or less [58]) and heavy smokers (defined as consuming 20 cigarettes or more [58]) was impossible. Future studies should consider classifying light, moderate, and heavy smokers to gain a better understanding of its associations with cardiovascular health factors. Association between smoking and central adiposity was also reported [59], and such association may be dose dependent [60]. The present study suggested that smoking cessation might not necessarily increase weight gain, a common concern about smoking cessation [61]. Regardless of the association between smoking and weight status, many adverse effects of smoking on metabolic syndrome, insulin resistance, and central adiposity [57,62] were sufficient justification for smoking cessation.

Inconsistent with current literature [63,64], association between vegetable and fruit intake and cardiovascular health factors were not found. This may be because of the imbalance in groups (only 47 subjects who consumed sufficient amount of vegetables and fruits versus 578 subjects who did not). Despite the lack of association found with cardiovascular health factors, it is noteworthy that 93% of the workers reported low vegetable and fruit intake. In addition to reducing cardiovascular diseases, vegetable and fruit intake reduced all-cause mortality and risk of cancers [64,65]. Therefore, future studies should confirm the associations between vegetable and fruit intake and cardiovascular health factors among construction workers and should consider promoting vegetable and fruiting intake among them.

This study had several limitations. Firstly, because of incomplete information, only two-thirds of the subjects were included in the analysis. As the data collection was conducted from 10:00 a.m. to 1:00 p.m., a high percentage of the subjects had random rather than fasting blood glucose tests, and hence had to be excluded in the final analysis. Besides, some dietary items used in this study were different from the existing recommendations of the American Heart Association [11] due to the cultural differences in dietary habits, for example, whole grain intake. Last, but not least, the validity of the questionnaire on health behaviors was not established. However, it was adopted from a validated questionnaire [29] and the data manipulation was as conservative as possible in order to minimize the over-estimation of the prevalence of health behaviors. Moreover, the questionnaire was reliable (test-retest). In terms of strengths, this study evaluated the cardiovascular health among construction workers systematically, using evidence-based cardiovascular health metrics [11], and provided baseline information for future surveillance, as well as insight to develop long-term strategies to promote cardiovascular health in the construction industry.

## 5. Conclusions

Approximately two-thirds of the construction workers achieved only three out of the seven “ideal” cardiovascular health indicators. The ideal fish and seafood consumption was associated with (1) ideal weight status and (2) ideal cholesterol level, whereas less soft drink consumption was associated with ideal total cholesterol level. The findings highlighted the importance of promoting cardiovascular health in the construction industry and provided some insights for future interventions, which should include increasing fish and seafood intake, decreasing soft drink consumption, and enhancing health literacy among older and less educated construction workers.

## Figures and Tables

**Table 1:** Sample descriptive.

Demographic, Cardiovascular Health Factors, and Health Behaviors	*n*	%
Gender		
Male	564	90.2
Female	61	9.8
Ethnicity		
Chinese—born in Hong Kong or have lived more than seven years in Hong Kong	526	84.0
Chinese—born in Mainland China	77	12.3
Minority	23	3.7
Education		
No Formal Education/Primary Education	135	21.7
Junior Secondary Education	249	40.0
Senior Secondary Education	170	27.3
Post-secondary Education (Diploma/Associate Degree/Degree etc.)	69	11.1
Age		
20–29	94	15.0
30–39	112	17.9
40–49	158	25.2
50–59	170	27.2
60 or older	92	14.7
Weight status		
Intermediate/Poor (body mass index (BMI) ≥ 23 kg/m^2^)	386	61.9
Ideal (BMI < 2323 kg/m^2^)	238	38.1
Blood cholesterol		
Intermediate/Poor (≥5.2 mmol/L or 200 mg/dL)	202	32.3
Ideal (<5.2 mmol/L or 200 mg/dL)	424	67.7
Blood pressure		
Intermediate/Poor (≥120 mm Hg/≥80 mm Hg)	484	81.3
Ideal (<120 mm Hg/<80 mm Hg)	111	18.7
Blood glucose		
Intermediate/Poor (≥5.6 mmol/L or 100 mg/dL)	49	7.8
Ideal (<5.6 mmol/L or 100 mg/dL)	577	92.2
Smoking		
Not desired (Smokers)	251	40.1
Ideal (Non-smoker)	375	59.9
Physical activity		
Not desired (<150 min moderate/week)	452	72.2
Ideal (≥150 min moderate/week)	174	27.8
Fruit and vegetable		
Not desired (<4.5 cups/day)	579	92.5
Ideal (≥4.5 cups/day)	47	7.5
Seafood or fish		
Not desired (<2 servings/week)	257	41.1
Ideal (≥2 servings/week)	369	58.9
Sweetened-beverage		
Not desired (≥36 oz/week)	169	27.0
Ideal (<36 oz/week)	457	73.0
	Mean	SD
Cardiovascular health score (0–7)	3.07	1.14
Number of ideal cardiovascular outcomes (0–4)	2.16	0.91
Number of ideal lifestyle indicators (0–3)	0.91	0.70

**Table 2:** Cardiovascular health outcomes.

Demographic Factors	Weight Status					Blood Cholesterol					Blood Pressure					Blood Glucose				
	Intermediate/Poor (BMI ≥ 23 kg/m^2^)		Ideal (BMI < 23 kg/m^2^)			Intermediate/Poor (≥5.2 mmol/L or 200 mg/dL)		Ideal (<5.2 mmol/L or 200 mg/dL)			Intermediate/Poor (≥120 mm Hg/≥80 mm Hg)		Ideal (<120 mm Hg/<80 mm Hg)			Intermediate/Poor (≥5.6 mmol/L or 100 mg/dL)		Ideal (<5.6 mmol/L or 100 mg/dL)		
	*n*	%	*n*	%	* p	*n*	%	*n*	%	* p	*n*	%	*n*	%	* p	*n*	%	*n*	%	* p
Gender																				
Male	350	62.2	213	37.8	0.63	184	32.6	380	67.4	0.62	440	82.1	96	17.9	0.14	45	8.0	519	92.0	0.70
Female	36	59.0	25	41.0		18	29.5	43	70.5		43	74.1	15	25.9		4	6.6	57	93.4	
Age																				
20–29	44	46.8	50	53.2	0.01	12	12.8	82	87.2	<0.001	71	77.2	21	22.8	0.43	1	1.1	93	98.9	<0.001
30–39	68	61.3	43	38.7		37	33.0	75	67.0		83	81.4	19	18.6		1	0.9	111	99.1	
40–49	110	69.6	48	30.4		52	32.9	106	67.1		118	79.2	31	20.8		14	8.9	144	91.1	
50–59	105	62.1	64	37.9		70	41.2	100	58.8		141	86.0	23	14.0		21	12.4	149	87.6	
60 or older	59	64.1	33	35.9		31	33.7	61	66.3		71	80.7	17	19.3		12	13.0	80	87.0	
Ethnicity																				
Chinese—Born in Hong Kong or have lived in Hong Kong for more than 7 years	325	61.9	200	38.1	0.23	179	34.0	347	66.0	0.10	417	81.8	93	18.2	0.44	45	8.6	481	91.4	0.22
Chinese—From Mainland	44	57.1	33	42.9		18	23.4	59	76.6		52	76.5	16	23.5		4	5.2	73	94.8	
Minority	17	77.3	5	22.7		5	21.7	18	78.3		15	88.2	2	11.8		0	0.0	23	100.0	
Education																				
No Formal Education/Primary Education	90	67.2	44	32.8	0.34	43	31.9	92	68.1	0.18	110	84.0	21	16.0	0.63	14	10.4	121	89.6	0.50
Junior Secondary Education	156	62.7	93	37.3		79	31.7	170	68.3		194	81.5	44	18.5		19	7.6	230	92.4	
Senior Secondary Education	98	57.6	72	42.4		64	37.6	106	62.4		129	80.6	31	19.4		13	7.6	157	92.4	
Post-secondary Education	40	58.0	29	42.0		16	23.2	53	76.8		48	76.2	15	23.8		3	4.3	66	95.7	

* *p*-values from chi-square tests.

**Table 3:** Lifestyle behaviors.

Demographic Factors	Smoking					Physical Activity					Fruit and Vegetable					Seafood or Fish					Soft Drink				
	Not Desired (Smokers)		Ideal (Non-smoker)			Not Desired (<150 min Moderate/Week)		Ideal (≥150 min Moderate/Week)			Not Desired (<4.5 Cups/Day)		Ideal (≥4.5 Cups/Day)			Not Desired (<2 Servings /Week)		Ideal (≥2 Servings /Week)			Not Desired (<2 Servings /Week)		Ideal (≥2 Servings /Week)		
	*n*	%	*n*	%	* *p*	*n*	%	*n*	%	* *p*	*n*	%	*n*	%	* *p*	*n*	%	*n*	%	* *p*	*n*	%	*n*	%	* *p*
Gender																									
Male	249	44.1	315	55.9	<0.001	398	70.6	166	29.4	0.007	524	92.9	40	7.1	0.22	238	42.2	326	57.8	0.056	163	28.9	401	71.1	0.001
Female	2	3.3	59	96.7		53	86.9	8	13.1		54	88.5	7	11.5		18	29.5	43	70.5		5	8.2	56	91.8	
Age																									
20–29	35	37.2	59	62.8	0.50	50	53.2	44	46.8	<0.001	88	93.6	6	6.4	0.54	56	59.6	38	40.4	<0.001	42	44.7	52	55.3	<0.001
30–39	47	42.0	65	58.0		73	65.2	39	34.8		103	92.0	9	8.0		55	49.1	57	50.9		56	43.4	73	56.6	
40–49	67	42.4	91	57.6		112	70.9	46	29.1		150	94.9	8	5.1		60	38.0	98	62.0		50	31.6	108	68.4	
50–59	72	42.4	98	57.6		142	83.5	28	16.5		153	90.0	17	10.0		61	35.9	109	64.1		29	17.1	141	82.9	
60 or older	30	32.6	62	67.4		75	81.5	17	18.5		85	92.4	7	7.6		25	27.2	67	72.8		9	9.8	83	90.2	
Ethnicity																									
Chinese—Born in Hong Kong or have lived in Hong Kong for more than 7 years	218	41.4	308	58.6	0.21	369	70.2	157	29.8	0.028	481	91.4	45	8.6	0.068	204	38.8	322	61.2	0.002	148	28.1	378	71.9	0.080
Chinese—From Mainland	27	35.1	50	64.9		63	81.8	14	18.2		75	97.4	2	2.6		36	46.8	41	53.2		13	16.9	64	83.1	
Minority	6	26.1	17	73.9		20	87.0	3	13.0		23	100.0	0	0.0		17	73.9	6	26.1		8	34.8	15	65.2	
Education																									
No Formal Education/Primary Education	48	35.6	87	64.4	0.002	113	83.7	22	16.3	<0.001	123	91.1	12	8.9	0.93	40	29.6	95	70.4	0.002	27	20.0	108	80.0	0.031
Junior Secondary Education	121	48.6	128	51.4		182	73.1	67	26.9		231	92.8	18	7.2		106	42.6	143	57.4		62	24.9	187	75.1	
Senior Secondary Education	63	37.1	107	62.9		118	69.4	52	30.6		158	92.9	12	7.1		71	41.8	99	58.2		54	31.8	116	68.2	
Post-secondary Education	18	26.1	51	73.9		36	52.2	33	47.8		64	92.8	5	7.2		39	56.5	30	43.5		25	36.2	44	63.8	

* *p*-values from chi-square tests.

**Table 4:** Overall cardiovascular health.

Demographic Factors	Cardiovascular Health Score			Ideal Health Behaviors			Ideal Cardiovascular Health Factors		
	Mean	SD	*p*	Mean	SD	*p*	Mean	SD	*p*
Gender ^a^									
Male	3.03	1.15	0.003	0.88	0.72	<0.001	2.14	0.92	0.21
Female	3.48	1.03		1.18	0.43		2.30	0.88	
Age ^b^									
20-29	3.73	1.13	<0.001 *	1.12	0.76	0.016 **	2.62	0.86	<0.001 *
30-39	3.16	1.11		0.95	0.71		2.21	0.87	
40-49	2.96	1.11		0.88	0.68		2.08	0.92	
50-59	2.79	1.09		0.81	0.69		1.98	0.92	
60 or older	2.99	1.06		0.91	0.66		2.08	0.85	
Ethnicity ^b^									
Chinese—Born in Hong Kong or have lived in Hong Kong for more than 7 years	3.06	1.15	0.55	0.93	0.71	0.61	2.13	0.92	0.14
Chinese—From Mainland	3.19	1.12		0.84	0.63		2.35	0.93	
Minority	2.96	0.88		0.87	0.63		2.09	0.67	
Education ^b^									
No Formal Education/Primary Education	2.92	1.14	<0.001 ^	0.86	0.69	<0.001 ^	2.06	0.84	0.17
Junior Secondary Education	2.97	1.01		0.82	0.67		2.16	0.87	
Senior Secondary Education	3.12	1.18		0.97	0.69		2.15	1.01	
Post-secondary Education	3.61	1.34		1.25	0.76		2.36	0.94	

^a^ Independent sample t-test was used to compared cardiovascular health score, ideal health behaviors, ideal cardiovascular health factors between male and female workers. ^b^ One-way analysis of variance (ANOVA) was used to compared cardiovascular health score, ideal health behaviors, and ideal cardiovascular health factors among educational, age, and ethnic groups. * significant difference between 20–29 and 60 or older; sig dif between 20–29 and 50–59; sig dif between 20–29 and 40–49; sig dif between 20–29 and 30–39. ** sig dif between 20–29 and 60 or older. ^ sig dif between post-sec and pri; sig dif between post-sec and jun sec; sig dif between post-sec and sen sec.

**Table 5:** Associations between health behaviors and cardiovascular health factors (logistic regressions) ^a^. CI—confidence interval.

Health Behaviors	Weight Status ^#^		*p*					Total Cholesterol ^#^		*p*	Adj. OR **	95% CI	*p*
	Crude OR *	95% CI		Adj. OR **	95% CI		*p*	Crude OR *	95% CI				
Smoking ^	1.52	(1.20, 1.92)	<0.001	1.36	(1.02, 10.81)		0.034	1.20	(0.93, 1.54)	0.15	1.28	(0.94, 1.75)	0.11
Physical activity (PA) ^	0.97	(0.71, 1.32)	0.83	0.97	(0.70, 10.33)		0.83	1.20	(0.86, 1.69)	0.28	1.24	(0.88, 1.74)	0.22
Fruit and vegetable ^	1.09	(0.71, 1.69)	0.69	0.98	(0.59, 10.63)		0.94	1.09	(0.71, 1.69)	0.69	0.82	(0.48, 1.42)	0.48
Fish and seafood ^	1.39	(1.11, 1.74)	<0.001	1.34	(1.02, 10.76)		0.038	1.20	(0.94, 1.54)	0.14	1.35	(1.00, 1.82)	0.048
Softdrink ^	1.12	(0.88, 1.44)	0.35	1.16	(0.85, 10.58)		0.36	1.30	(1.00, 1.69)	0.049	1.48	(1.06, 2.07)	0.020
	**Blood Pressure ^#^**		***p***	Adj. OR **	**95% CI**		***p***	**Blood Glucose ^#^**		***p***	Adj. OR **	**95% CI**	***p***
	Crude OR *	**95% CI**						Crude OR *	**95% CI**				
Smoking ^	0.97	(0.70, 1.36)	0.87	1.05	(0.70, 1.58)		0.83	1.13	(0.66, 1.91)	0.66	0.86	(0.46, 1.59)	0.62
PA ^	0.81	(0.53, 1.25)	0.35	0.80	(0.52, 1.23)		0.31	1.41	(0.72, 2.77)	0.32	1.43	(0.72, 2.81)	0.30
Fruit and vegetable ^	0.77	(0.44, 1.35)	0.36	0.64	(0.34, 1.21)		0.17	0.79	(0.27, 2.27)	0.66	1.10	(0.37, 3.29)	0.86
Fish and seafood ^	1.00	(0.73, 1.38)	0.98	0.95	(0.64, 1.40)		0.79	0.99	(0.58, 1.67)	0.96	1.06	(0.56, 2.01)	0.85
Softdrink ^	0.91	(0.65, 1.29)	0.60	0.84	(0.55, 1.30)		0.43	0.89	(0.48, 1.64)	0.70	1.07	(0.50, 2.30)	0.86

^a^ logistic regression was used to examine associations between health behaviors and cardiovascular health factors adjusting for cluster effects. ^ Smoking: non-smokers vs. smokers (ref); PA: ≥150 min moderate/week vs. <150 min moderate/week (ref); Fruit and vegetable: ≥4.5 cups/day vs. <4.5 cups/day (ref); Fish and seafood: ≥2 servings/week vs. <2 servings/week (ref); Soft drink: ≥2 servings/week vs. <2 servings/week (ref); ^#^ Weight status: body mass index (BMI) < 23 kg/m^2^ vs. BMI ≥ 23 kg/m^2^ (ref); Blood cholesterol: <5.2 mmol/L or 200 mg/dL vs. ≥5.2 mmol/L or 200 mg/dL (ref); Blood pressure: <120 mm Hg/<80 mm Hg vs. ≥120 mm Hg/≥80 mm Hg (ref); Blood glucose: <5.6 mmol/L or 100 mg/dL vs. ≥5.6 mmol/L or 100 mg/dL (ref). * crude OR was adjusted for age, gender, ethnicity, and education. ** adjusted OR was adjusted for age, gender, ethnicity, and education and other lifestyle behaviors.

## Appendix A

**Table A1:** Health Behaviors’ Measurement.

Variables	Questions	Response Options	Test-Retest Reliability (Spearmen’s Correlation Coefficient)	“Ideal” vs. “Undesired” Classification Agreement (Kappa)
Dietary pattern	3. How frequently you eat the following food (e.g., soft drinks, vegetables, fruits, fish and seafood) in the last month? (Frequency)	1. never, 2. 1–3 days a month, 3. one day a week, 4. two days a week, 5. three days a week, 6. four days a week, 7. five days a week, 8. six days a week, 9. seven days a week	0.812 (seafood and fish)–0.933 (vegetable)	0.818 (seafood and fish), 0.846 (vegetable and fruits) and 1 (soft drinks)
	2. How much did you eat the following food in the day that you eat it? (Amount)	1. 0 serving, 2. 1 serving, 3. 2 servings, 4. 3 servings, 5. 4 servings, 6. 5 servings, 7. 6 servings, or 8. 7 servings or more	0.782 (seafood and fish)–1 (soft drinks)	
Smoking	1. How frequently did you smoke in the last month? (Frequency)	1. never, 2. 1–3 days a month, 3. one day a week, 4. two days a week, 5. three days a week, 6. four days a week, 7. five days a week, 8. six days a week, 9. orseven days a week	1	1
	2. How much did you smoke in the day that you smoke?” (Amount)	1. none, 2. 1 cigarette, 3. 2 cigarettes, 4. 3 cigarettes, 5. 4 cigarettes, 6. 5 cigarettes or more	1	
Physical activity	1. In the last month, how frequently did you participate in sport? (Frequency)	1. never, 2. 1–3 days a month, 3. one day a week, 4. two days a week, 5. three days a week, 6. four days a week, 7. five days a week, 8. six days a week, 9. seven days a week	0.981	0.762
	2. How long did you participate in sport on the day that you participated in sport? (Duration)	1. less than 30 minutes, 2. more than 30 minutes but less than 1 hour, 3. 1 hour but less than 2 hours, 4. 2 hours but less than 3 hours, 5. 3 hours but less than 4 hours, 6. 4 hours or more	0.931	
	3. Which most common sport did you play? (Type)	1. ball games, 2. cycling, 3. running, 4. jogging, 5. swimming, 6. hiking, or 7. others	0.943	
